# Arm or Leg? The best site for injections in pediatric patients

**DOI:** 10.3389/fped.2023.1323337

**Published:** 2023-12-20

**Authors:** Kennedy A. Sabharwal, Michael W. Simon

**Affiliations:** ^1^ College of Medicine, University of Kentucky, Lexington, KY, United States; ^2^ Department of Pediatrics, University of Kentucky, Lexington, KY, United States

**Keywords:** DTaP vaccine, DTaP, diphtheria, tetanus and acellular pertussis vaccine, pediatric vaccination, deltoid, anterolateral thigh

Infants, toddlers, children, and even adults receive injections for preventive health and illness. The current rule of thumb for the site of injections for children is that for those of two years of age and younger to administer injections in their anterolateral thigh since the deltoid area is still maturing and involved in the brachial plexus development (1). After three years of age, there may be a transition to administering injections in the deltoid area. For adults and children of the appropriate age, it is much easier to roll up your sleeve than drop your pants for an injection. However, the anterolateral thigh may remain the best option and site for injections even for those older than two years of age.

Pain after injection is the most frequent complaint when using the deltoid injection site (2). The pain response in infants may be quantified by monitoring heart rate, blood pressure, and breathing changes and has been noted to be similar for infants with injections in the deltoid and vastus lateralis muscle group (3). Due to the extent of their nervous system development, their perception and processing of painful stimuli may be more dull than acute. However, it was observed that the duration of crying in pediatric patients was statistically shorter with injections in the vastus lateralis compared to the deltoid muscle (3). Because of the function of maintaining our upright position, the thigh has a much larger muscle mass compared to the arm and a greater number of motor neurons compared to somatic pain-perceiving neurons. In adults, the thickness of the deltoid muscle ranges between 0.15–1.40 cm depending on which area of the muscle you are measuring (4), compared to around 2.41 cm mean thickness of the vastus lateralis (anterolateral thigh) muscle in adults (5). A greater muscle mass for intramuscular injections optimizes the immunogenicity of vaccines (6). The larger muscle mass may also produce a buffering effect reducing the level of somatic nerve pain. Additionally considering function, the thigh has gross motor function compared to the arm and hand, which have fine motor activity.

Specific information has been reported with the acellular Diphtheria-Tetanus-Pertussis (DPT) vaccination comparing pain in the deltoid vs. vastus lateralis muscle groups (3, 7, 8). In multiple studies in children between one and six years of age, less pain was noted with injections given in the thigh over the deltoid. For those 4 to 6 years of age, the fifth DPT dose produced less local reaction when given in the thigh vs. the deltoid region. Although research has been conducted to show that antibody responses to the DPT vaccine do not differ when administered over the deltoid vs. the anterolateral thigh, it has been reported that the DTP-containing vaccinations are better tolerated when administered at the anterolateral thigh and produce fewer local reactions (9).

In the early months of life, the vastus lateralis is recommended as the injection site since the deltoid muscle mass is insufficient for injections and may also cause nerve damage (10). Compared to the vastus lateralis, the deltoid muscle contains many nearby blood vessels and nerves. This increases the risk of intramuscular injections at this site causing nerve damage. Comparatively, the vastus lateralis muscle is free of major blood vessels and nerves which reduces the potential for damage when used as the injection site. the size of the muscle and nerve density at the injection site are important factors for the perception of pain and discomfort (3). This would carry over for this being the recommended site for injections in young children and the preferred site for any injection at any age. Muscle mass has been shown to deteriorate with age, thus, this may be another important consideration for injections in the anterior lateral thigh for the elderly population (11).

Considering the potential for nerve damage, injury, and adverse reactions, the anterolateral thigh should be considered as a preferred site for vaccinations and injections for all aged individuals.
